# Rare Genetic Disorders: Novel Treatment Strategies and Insights Into Human Biology

**DOI:** 10.3389/fgene.2021.714764

**Published:** 2021-08-06

**Authors:** Peter J. Koch, Maranke I. Koster

**Affiliations:** Department of Anatomy and Cell Biology, Brody School of Medicine (BSOM) at East Carolina University (ECU), Greenville, NC, United States

**Keywords:** TP63, ectodermal dysplasia, orphan (rare) diseases, rare genetic disorders, gene therapy, protein therapy, iPSC disease modeling

## Abstract

The last decade has seen a dramatic increase in innovative ideas for the treatment of genetic disorders for which no curative therapies exist. Gene and protein replacement therapies stand out as novel approaches to treat a select group of these diseases, such as certain tissue fragility disorders. Further, the advent of stem cell approaches, such as induced pluripotent stem cells (iPSC) technology, has led to the development of new methods of creating replacement tissues for regenerative medicine. This coincided with the discovery of genome editing techniques, which allow for the correction of disease-causing mutations. The culmination of these discoveries suggests that new and innovative therapies for monogenetic disorders affecting single organs or tissues are on the horizon. Challenges remain, however, especially with diseases that simultaneously affect several tissues and organs during development. Examples of this group of diseases include ectodermal dysplasias, genetic disorders affecting the development of tissues and organs such as the skin, cornea, and epithelial appendages. Gene or protein replacement strategies are unlikely to be successful in addressing the multiorgan phenotype of these diseases. Instead, we believe that a more effective approach will be to focus on correcting phenotypes in the most severely affected tissues. This could include the generation of replacement tissues or the identification of pharmaceutical compounds that correct disease pathways in specific tissues.

## Challenges Associated With Research Into Rare Genetic Diseases

One of the most rewarding aspects of biomedical research is the ability to gain basic biological knowledge by understanding disease processes, and to use this knowledge to develop new therapies. One area of particular interest is the field of rare genetic diseases. From a biologist’s point of view, rare diseases represent an “experiment of nature,” as outlined by Sir Archibald Garrod in his 1928 speech to the Medical Society of London entitled: “The Lessons of Rare Maladies (1928).” Almost two centuries earlier, in 1657, the renowned physician and anatomist Dr. William Harvey already recognized the importance of studying rare disorders and stated: *“Nature is nowhere accustomed more openly to display her secret mysteries than in cases where she shows tracings of her workings apart from the beaten paths; nor is there any better way to advance the proper practice of medicine than to give our minds to the discovery of the usual law of nature, by careful investigation of cases of rarer forms of disease”* (Harvey et al., 1847).

Current definitions of what constitutes a rare disease differ by country or region. In the United States, rare diseases are defined as those affecting less than 200,000 people, whereas in the European Union rare diseases are defined as those that affect less than 1 in 2,000 people. It is estimated that over 7,000 rare diseases affect between 25 and 30 million people in the United States, and approximately 300 million people worldwide (Nguengang Wakap et al., 2020). The molecular and cellular pathologies underlying many of these diseases are not known. Further, due to the small number of patients affected by each disease, there is little motivation for commercial entities to invest in research and treatment development for these rare diseases, often referred to as “orphan diseases.”

The small number of patients and wide geographical distribution of patients render clinical trials difficult. In addition, clinical expertise in rare disorders is sometimes limited, as most physicians will rarely encounter multiple individuals affected by the same rare disease. These factors have hindered the development and testing of new therapies. Nevertheless, research into rare diseases has accelerated with the development of novel approaches in personalized medicine, such as genome editing, gene and cell therapy, and tissue regeneration.

## Ectodermal Dysplasias: A Group of Rare Genetic Disorders

**FIGURE 1:**
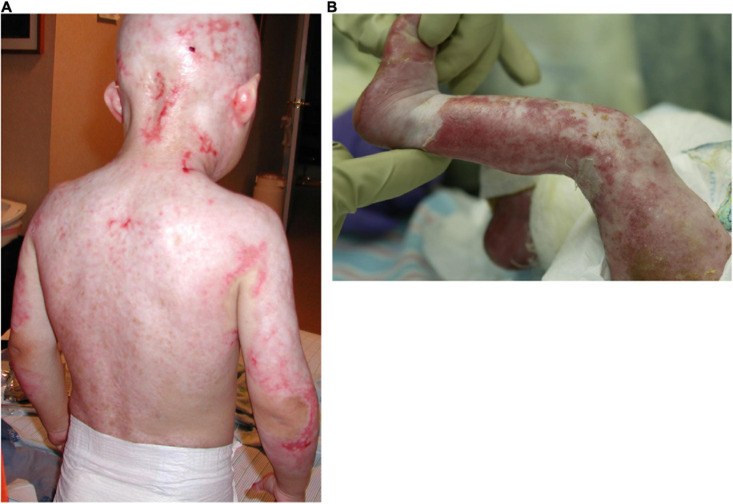
Severe skin erosions on the back **(A)** and the leg **(B)** of two AEC patients.

Ectodermal dysplasias are a group of rare genetic disorders characterized by developmental abnormalities in ectodermal derivatives (Wright et al., 2019). Here, we will focus on two ectodermal dysplasias that are caused by missense mutations in the TP63 gene: ectrodactyly, ectodermal dysplasia, and cleft lip/palate syndrome [EEC; OMIM #604292] and ankyloblepharon-ectodermal defects-cleft lip/palate syndrome [AEC; OMIM #106260] (Rinne et al., 2007). The TP63 transcription factor is essential for the development, maintenance, and regeneration of stratified epithelia and their appendages (Koster, 2010). Thus, it is perhaps unsurprising that individuals affected by AEC and EEC show abnormalities in a wide range of ectodermal derivatives, including skin, hair, cornea, teeth, and limbs (Rinne et al., 2007). Despite many similarities in clinical symptoms in AEC and EEC, notable differences also exist. For example, skin erosions are more prevalent in AEC (Siegfried et al., 2005; Julapalli et al., 2009; Maillard et al., 2019; Figure 1), although they can occur in both disorders. Corneal lesions and visual impairment are more commonly observed in EEC (Di Iorio et al., 2012; Felipe et al., 2012). Phenotypical differences between EEC and AEC are likely linked to the site of the TP63 mutation, which is generally located in the DNA binding domain in EEC and in the SAM protein interaction domain in AEC (Celli et al., 1999; McGrath et al., 2001; Rinne et al., 2007). The presence of a unique TP63 mutation in each patient as well as the fact that multiple different tissues are affected, present major challenges in the design of rational therapies for these patients. As the skin and eye represent the most severely affected organs in AEC and EEC patients, we will focus on these organs here.

### Skin Erosions

Skin erosions occur in the majority of AEC patients and can cover as much as 80% of the body surface (Vanderhooft et al., 1993; Siegfried et al., 2005; Julapalli et al., 2009; Maillard et al., 2019). Lesions are characteristically localized to the scalp, but often involve additional body sites, including the trunk and the extremities. These skin erosions cause intense pain, frequently interfere with daily activities and medical procedures, and require constant care. Further, the resulting skin barrier defects place individuals at high risk for local as well as systemic infections, sometimes with life-threatening consequences. Although the histology of very few lesional skin biopsies has been published, the lesions appear to involve blister formation, a disintegration of the epidermis, and a failure of the epidermis to properly adhere to the underlying dermis (Payne et al., 2005; Zhang et al., 2019). Nevertheless, because of the relative paucity of skin samples, little is known regarding the progression of histopathological changes associated with skin fragility in AEC. We previously analyzed a set of 23 peri-lesional AEC skin biopsies and found that AEC skin exhibited focal loss of several desmosomal and hemidesmosomal proteins that have previously been linked to skin fragility (Koster et al., 2009; Tsuruta et al., 2011; Petrof et al., 2012; Koster et al., 2014; Dinella et al., 2018). Similar observations were made by others using smaller sets of skin biopsies or primary keratinocytes obtained from patient skin (Clements et al., 2012; Zarnegar et al., 2012; Ferone et al., 2013; Aberdam et al., 2020). Mechanistically, these findings have been confirmed in an *in vitro* stem cell-based disease model described below (Dinella et al., 2018).

### Corneal Abrasions/Limbal Stem Cell Deficiency

EEC patients have multiple abnormalities affecting the eye, often culminating in impaired vision (Di Iorio et al., 2012; Felipe et al., 2012). The primary cause for the loss of vision is believed to be limbal stem cell deficiency (LSCD). Limbal stem cells reside at the periphery of the cornea in a region termed the limbus, and are responsible for maintaining and regenerating the corneal epithelium (Osei-Bempong et al., 2013). In addition to harboring limbal stem cells, the limbus also acts as a physical barrier between the conjunctiva and cornea, preventing conjunctival cells from migrating into the cornea under normal conditions. In EEC patients, the limbal stem cells malfunction and ultimately disappear. As a consequence, the limbal barrier is compromised leading to corneal conjunctivalization, and subsequent loss of vision (Di Iorio et al., 2012; Felipe et al., 2012). The progression of this condition is likely exacerbated by structural abnormalities of the corneal epithelial cells as well as the absence or malfunction of eye-associated glands that normally lubricate the cornea (Di Iorio et al., 2012; Felipe et al., 2012). Although LSCD develops in most EEC patients, the precise role of mutant TP63 proteins in causing this abnormality is not known. Based on its function in other types of stem cells, it is likely that TP63 might function to control stem cell maintenance, proliferation, differentiation and/or migration. *In vitro* and *in vivo* models will be essential to dissect out the pathophysiological role of mutant TP63 proteins and to develop novel therapeutic approaches.

## New Therapeutic Approaches for Rare Genetic Disorders

In recent years, considerable advances have been made in the development of effective therapeutics for genetic skin disorders and ectodermal dysplasias. One of the most striking examples is the use of a prenatal protein replacement strategy for the treatment of X-linked hypohidrotic ectodermal dysplasia (XLHED), an ectodermal dysplasia caused by loss-of-function mutations in the EDA gene (Schneider et al., 2018). In addition to dental and hair abnormalities, patients with XLHED are at high risk of developing life-threatening hyperthermia due to the absence of functional sweat glands (Fete et al., 2014). Administering the missing ligand EDA *in utero* led to a complete restoration of sweat gland number and function in humans (Schneider et al., 2018). This is the first example of a successful prenatal treatment of a rare genetic disorder, and opens the door for similar treatments for other genetic disorders. Specifically, protein replacement strategies are uniquely suited for therapeutic development for disorders caused by the absence of a ligand. In addition, strategies that rely on restoring expression of missing or mutated proteins have also been used for the treatment of the skin blistering disorders Junctional Epidermolysis Bullosa (JEB) and Recessive Dystrophic Epidermolysis Bullosa (RDEB), caused by mutations in extracellular components of the basement membrane. In these examples, transgenic autologous keratinocytes overexpressing the missing or mutated proteins were grafted onto patients affected by these disorders leading to the long-term formation of normal skin (Hirsch et al., 2017; Eichstadt et al., 2019).

In some rare genetic disorders, nature has provided us with tools to overcome disease phenotypes. The prime example of this is revertant mosaicism, which occurs in a subset of genetic skin disorders, including RDEB and JEB (Lim et al., 2017). As a result of revertant mosaicism, patients develop patches of healthy skin in which cells have spontaneously regained a wild type genotype (i.e., eliminated a mutant allele). These cells can subsequently be isolated, expanded, and used to transplant onto the patient (Gostynski et al., 2014; Matsumura et al., 2019).

## Potential Therapies for AEC and EEC

Revertant mosaicism has not been described for AEC in the literature. However, AEC patients have described focal outgrowth of islands of normal-appearing skin that ultimately led to permanent healing of the skin erosions (personal communications). This observation warrants further scientific exploration. If these islands indeed represent revertant patches of skin, then a skin treatment based on the expansion and grafting of such revertant cells (or gene-corrected patient keratinocytes; see below) might be feasible for AEC patients in the future.

Treatment strategies based on restoring wild type TP63 function to mutant TP63 proteins have also been proposed. Interestingly, it was found that the compound PRIMA-1 (MET) can restore transcriptional activity of some mutant forms of TP53, a homolog of TP63 (Bykov et al., 2002). In fact, PRIMA-1 (MET) is currently being tested in a phase III clinical trial for the treatment of certain myelodysplastic syndromes in which patients carry TP53 mutations, and is being explored for therapeutic use in other malignancies (Menichini et al., 2021). Based on structural similarities between TP53 and TP63, studies to investigate the potential for PRIMA-1 (MET) to restore the function of mutant TP63 proteins were conducted. It was found that PRIMA-1 (MET), can indeed restore some function to certain TP63 mutants (Shen et al., 2013). Further, PRIMA-1 (MET) treatment partially restored expression of some epidermal differentiation markers in AEC keratinocytes (Rokaeus et al., 2010; Shalom-Feuerstein et al., 2013). Based on these encouraging findings, two AEC patients were treated with PRIMA-1 (MET) leading to an improvement of the epidermal covering of lesions in these patients (Aberdam et al., 2020). In addition, the patients reported a dramatic reduction in pain leading to a termination of painkiller use, and an improvement in quality of life. However, although of clinical benefit, late epidermal differentiation and barrier function did not appear to be restored in the treated areas, and the mechanism of PRIMA-1 (MET) in this context remains unclear.

Another proposed mode of treatment for AEC and EEC is allele-specific silencing of the mutant TP63 allele. It has been demonstrated that siRNAs that target specific TP63-EEC mutations lead to some restoration of TP63 transcriptional activity *in vitro* (Barbaro et al., 2016; Novelli et al., 2016). Applying this approach to patients faces significant challenges, including the necessity of designing unique siRNAs for each disease-causing TP63 mutation, potential off-target effects of the siRNA, challenges related to siRNA delivery, and the presumably short-term effect of siRNAs on mutant TP63 silencing.

Finally, an approach that is being considered for several blistering skin disorders is cell therapy using gene-corrected keratinocytes (Sebastiano et al., 2014; Jackow et al., 2019). Cell therapy to address skin fragility in AEC patients represents a viable option, given that several laboratories have developed protocols to direct the differentiation of induced pluripotent stem cells (iPSC) into the keratinocyte lineage (Itoh et al., 2011; Petrova et al., 2014). This approach requires the generation of patient-specific iPSC, correction of the disease-causing TP63 mutation, and the subsequent differentiation of these cells into keratinocyte sheets for transplantation onto patients. Although feasible in principle, several drawbacks to this approach exist: this approach is extremely time-consuming and expensive, is technically very complex, and carries the inherent risk of introducing unintentional mutations into the genome of iPSC-derived cells. Further, no cell-based treatments based on the use of gene-corrected iPSC have been approved in the US, leading to an additional hurdle in developing and testing such therapies. Additional barriers to this approach include potential genetic heterogeneity of patient-derived iPSC, off-target effects of gene correction approaches, and variability of iPSC differentiation protocols. Consequently, a comprehensive set of generally accepted quality control measures to generate iPSC and iPSC-derived somatic cells for clinical applications will be required to make this approach a reality. Still, using gene-corrected iPSC-derived keratinocytes to treat painful and extensive skin lesions remains a promising option for future treatments of AEC and EEC patients. As outlined below, iPSC and iPSC-derived somatic patient cells can also be extremely useful in identifying disease pathways and in screening for compounds that can restore normal function of these pathways.

## Back to the Basics

Considering the lack of effective treatment options for these complex disorders, it is desirable to focus our efforts on the most severely affected tissues and organs. In the case of AEC and EEC, our focus has been on the skin for AEC and on the eye for EEC. The question to answer is how we can suppress disease phenotypes in these tissues, even if we currently lack the ability to correct the underlying defect in all affected tissues. One approach is to identify disease mechanisms in specific cell types and to develop tools, such as small compounds, to specifically interfere with disease pathways. To that end, we recently established iPSC-based *in vitro* disease models for AEC and EEC. iPSC lines were established from AEC and EEC patients. Using genome-editing tools, we corrected the TP63 mutation in each iPSC line, thereby creating pairs of cell lines that were identical except for the presence, or absence, of a TP63 mutation (Dinella et al., 2018). After *in vitro* differentiation of these cells into keratinocytes, several cellular defects were identified in patient (but not in gene-corrected) iPSC-derived keratinocytes. These included defects in cell adhesion and differentiation, phenotypes also observed in patient skin (Dinella et al., 2018). The directed differentiation of iPSC into other cells types affected by TP63 mutations will be useful to analyze additional disease mechanisms, such as limbal stem cell deficiency observed in EEC patients. As outlined above, these cell systems will be ideally suited to screen for therapeutics that can suppress defined disease pathways or that activate compensatory pathways.

Although we have come a long way in understanding complex diseases such as AEC and EEC, much remains to be learned about these diseases, and creative new therapies are needed to address the severe phenotypes associated with these disorders.

## Data Availability Statement

The original contributions presented in the study are included in the article/supplementary material, further inquiries can be directed to the corresponding author.

## Author Contributions

Both authors conceptualized and wrote the manuscript and approved the submitted version.

## Author Disclaimer

The views expressed in this article are those of the authors and not necessarily those of the NHS, the NIHR, or the Department of Health and Social Care.

## Conflict of Interest

The authors declare that the research was conducted in the absence of any commercial or financial relationships that could be construed as a potential conflict of interest.

## Publisher’s Note

All claims expressed in this article are solely those of the authors and do not necessarily represent those of their affiliated organizations, or those of the publisher, the editors and the reviewers. Any product that may be evaluated in this article, or claim that may be made by its manufacturer, is not guaranteed or endorsed by the publisher.
